# Economic evaluation of including the EV71 vaccine in the national immunization program: a modeling study in Guangxi, China

**DOI:** 10.3389/fpubh.2026.1842766

**Published:** 2026-06-29

**Authors:** Quan He, Xiaoting Liao, Linlin Deng, Zhengqin Su, Weiwen Zhou, Yi Mo, Xiong Zou, Caifang Li, Yong Lv, Caitong He, Haibin Wei, Hai Li

**Affiliations:** 1 School of Public Health and Management, Guangxi University of Traditional Chinese Medicine, Nanning, China; 2 Key Laboratory of Integrated Traditional Chinese and Western Medicine Translational Medicine for High Incidence Infectious Diseases in Guangxi, Nanning, China; 3 Sterile Supply Department, The First Affiliated Hospital of Guangxi University of Traditional Chinese Medicine, Nanning, China; 4 Immunization Planning Institute, Guangxi Zhuang Autonomous Region Center for Disease Control and Prevention, Nanning, China; 5 Vaccine Clinical Research Institute, Guangxi Zhuang Autonomous Region Center for Disease Control and Prevention, Nanning, China; 6 The Maternal and Child Health Hospital of Guangxi Zhuang Autonomous Region, Nanning, China; 7 Department of Pediatrics, Liuzhou Maternal and Child Health Hospital, Liuzhou, China; 8 Yulin Maternal and Child Health Hospital, Yulin, China; 9 Development Planning Department, Guangxi University of Traditional Chinese Medicine, Nanning, China

**Keywords:** cost–benefit analysis, cost-effectiveness analysis, decision tree–Markov model, EV71, health economics, HFMD, national immunization program, vaccination policy

## Abstract

**Background:**

Hand, foot, and mouth disease (HFMD) remains a major public health concern among children in China, particularly in those under 5 years of age. Enterovirus 71 (EV71) is the leading cause of severe cases and mortality. Although EV71 vaccines are highly effective, they are currently self-paid in China, and evidence on publicly funded strategies remains limited. This study evaluated the health and economic impact of including the EV71 vaccine in the National Immunization Program (NIP) in Guangxi from a societal perspective, incorporating both direct medical costs and indirect productivity losses.

**Methods:**

A decision tree–Markov model simulated a cohort of 420,000 newborns over a 5-year horizon. Two strategies were compared: NIP inclusion (free vaccination) and the current self-paid approach. Cost-effectiveness and cost–benefit analyses were conducted, with outcomes including incremental cost-effectiveness ratio (ICER) and benefit–cost ratio (BCR). Sensitivity analyses were performed to assess uncertainty.

**Results:**

Compared with the self-paid strategy, NIP inclusion increased vaccination by 344,190 individuals and prevented 14,234 HFMD cases and 16 deaths. Disease-related costs decreased by USD 10.15 million, while vaccination costs increased by USD 19.71 million (BCR = 0.515). The intervention yielded 1,260.8 additional quality-adjusted life years (QALYs), with an ICER of USD 8,319.99/QALY, approximately 1.005 times the per capita GDP of Guangxi, indicating acceptable cost-effectiveness. Vaccine price was the most influential parameter; reducing the price to USD 14.94 per dose would achieve cost–benefit balance.

**Conclusions:**

Including the EV71 vaccine in the NIP would substantially reduce HFMD burden and is cost-effective. Price reductions could further improve economic outcomes and enable net societal benefits, supporting policy decisions in China and similar settings.

## 1 Introduction

Hand, foot, and mouth disease (HFMD) represents a significant public health concern in China and globally, particularly among children under 5 years of age. Since 2008, HFMD has been classified as a notifiable infectious disease in China, with more than 13 million reported cases, including severe and fatal outcomes, highlighting its substantial disease burden (1). Among the multiple causative pathogens, enterovirus 71 (EV71) is of particular concern due to its strong neurotropism and its close association with severe complications such as meningitis, encephalitis, and death.

Vaccination is widely recognized as the most effective strategy for preventing EV71-associated HFMD. Currently, three inactivated EV71 vaccines have been licensed in China, and clinical trials as well as follow-up studies have demonstrated their high efficacy and substantial protective effects (2). Real-world evidence and clinical data further indicate that these vaccines provide over 90% protection against EV71-associated HFMD and nearly 100% protection against severe and hospitalized cases (3). In addition, population-based studies have shown that the widespread use of EV71 vaccines can significantly reduce disease incidence, particularly among children under 5 years of age, thereby preventing a large number of cases (4).

Despite the availability of effective vaccines, EV71 vaccination in China is currently implemented on a voluntary, self-paid basis and has not been included in the National Immunization Program (NIP). This financing mechanism may limit vaccination coverage and lead to inequities in access, especially in economically less developed regions. Several provincial studies have reported relatively low vaccination coverage under the self-paid model, which may weaken the overall population-level impact of vaccination programs (5).

Guangxi Zhuang Autonomous Region, located in southwestern China, has experienced a substantial burden of HFMD according to regional epidemiological evidence (6). In addition, Guangxi reports approximately 420,000 live births annually, representing a large birth cohort that could potentially benefit from childhood immunization programs (7, 8). Despite the availability of effective EV71 vaccines, vaccination coverage remains constrained under the current self-paid financing model, particularly in areas with limited economic resources.

Economic evaluation is particularly important for informing vaccine financing decisions because the health and economic impacts of vaccination are highly context-specific. Disease incidence, healthcare utilization patterns, vaccine uptake, medical costs, and socioeconomic conditions vary substantially across provinces in China. Consequently, findings from previous economic evaluations conducted in other regions may not be directly transferable to Guangxi.

Although several studies have assessed the effectiveness and economic value of EV71 vaccination in China, evidence regarding the potential inclusion of EV71 vaccine in the National Immunization Program (NIP) remains limited at the provincial level, especially from a societal perspective that incorporates both direct medical costs and indirect productivity losses. As policymakers increasingly consider the expansion of publicly funded immunization programs, region-specific evidence is needed to evaluate whether the health benefits and economic returns of EV71 vaccination justify public investment in Guangxi.

Economic evaluation provides essential evidence for immunization policy decision-making. Cost-effectiveness analysis (CEA) and cost–benefit analysis (CBA) are key approaches for assessing the value of vaccination programs (9). Indicators such as the incremental cost-effectiveness ratio (ICER) and the benefit–cost ratio (BCR) offer complementary information for determining whether an intervention is cost-effective and whether its overall benefits outweigh its costs. Notably, analyses conducted from a societal perspective can capture both direct medical costs and indirect costs, such as productivity losses resulting from morbidity and premature mortality.

Therefore, this study aimed to evaluate the health and economic outcomes of including the EV71 vaccine in the NIP and to compare this strategy with the current self-paid vaccination approach in Guangxi, China. Using a decision tree–Markov hybrid model based on a birth cohort of 420,000 newborns, we estimated disease burden, costs, quality-adjusted life years (QALYs), and economic outcomes from a societal perspective. By generating region-specific evidence on both cost-effectiveness and cost–benefit performance, this study seeks to address an important evidence gap and provide quantitative support for future immunization policy decisions in Guangxi and other settings with similar epidemiological and socioeconomic characteristics.

## 2 Methods

### 2.1 Study design and analytical framework

This study employed a model-based health economic evaluation by developing a decision tree–Markov hybrid model to assess the economic value of different vaccination strategies for inactivated enterovirus 71 (EV71) vaccines among children in Guangxi, China. The model simulated the incidence, clinical progression, and healthcare resource utilization associated with HFMD under different vaccination strategies from a societal perspective with limited lifetime extrapolation, and compared both costs and health outcomes.

A decision tree was used to represent the initial vaccination strategies and dosing pathways (10, 11), while a Markov model was applied to simulate long-term transitions between health states in children (12, 13), enabling the estimation of long-term health benefits and economic outcomes.

### 2.2 Study population and setting

The study population consisted of a simulated closed cohort of 420,000 newborns in Guangxi Zhuang Autonomous Region in 2024. The cohort was followed over the study time horizon to estimate population-level outcomes under different vaccination strategies. Population data were obtained from the *Guangxi Statistical Yearbook* (7) and the *2024 Statistical Communiqué on National Economic and Social Development* (8), ensuring that the model reflects real-world demographic characteristics and supports population-level inference.

### 2.3 Comparator strategies

According to recommendations from the National Health Commission of China, the EV71 vaccine is currently classified as a non–National Immunization Program (non-NIP) vaccine (Category II vaccine) and is administered on a voluntary, self-paid basis. The recommended immunization schedule consists of two doses, with the first dose administered at 6 months of age and the second dose given one month later.

This study compared the following two vaccination strategies: **Current strategy (comparator group):** EV71 vaccination is not included in the NIP and is administered on a self-paid basis. **Intervention strategy (intervention group):** EV71 vaccination is included in the NIP, with vaccination costs fully covered by the government and vaccines provided free of charge.

Under both strategies, individuals were further categorized according to actual vaccination uptake into three dosing scenarios: no vaccination, one-dose vaccination, and two-dose vaccination (complete immunization schedule).

### 2.4 Model structure

**Figure 1:**
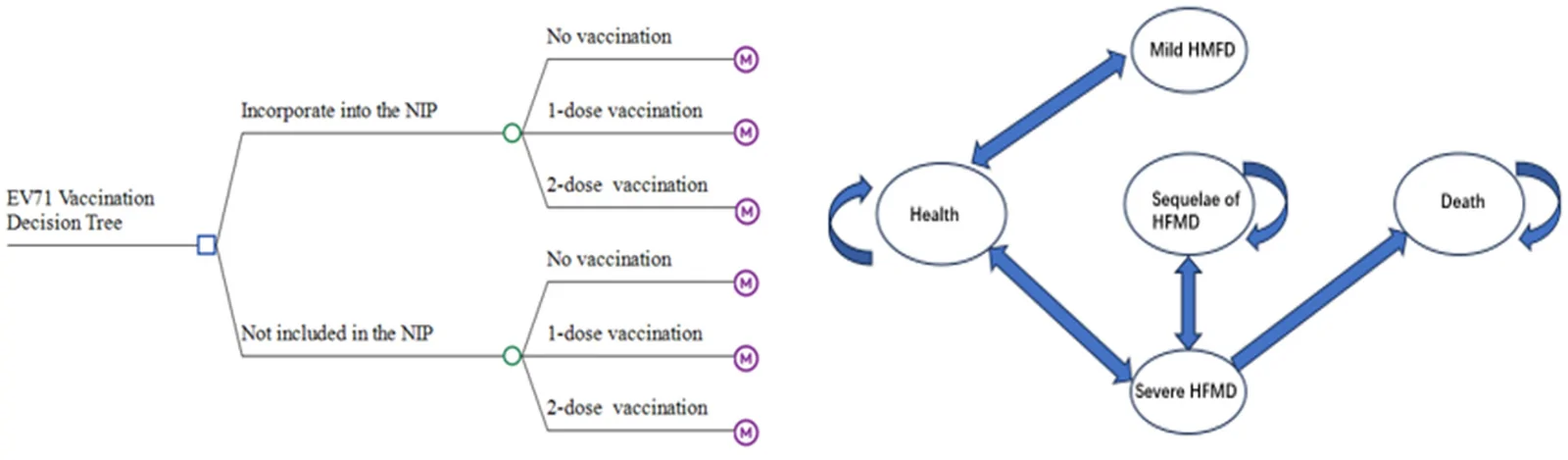
Decision tree–Markov model for EV71 vaccination strategies.

A decision tree–Markov hybrid model was constructed and implemented using R software (version 4.4.3). The overall model structure is illustrated in Figure 1.

At the initial stage, a decision tree was developed with the primary decision node defined as “inclusion in the National Immunization Program (NIP) or not,” followed by branches representing different vaccination dosing scenarios (0–2 doses). The terminal nodes of the decision tree were linked to a Markov model to simulate long-term disease progression.

The Markov model included the following health states: healthy, mild HFMD, severe HFMD, sequelae, HFMD-related death, and all-cause death. During each cycle, individuals transitioned between health states according to predefined transition probabilities. Each state was assigned corresponding cost and utility values, allowing for the estimation of cumulative health outcomes over time. As summarized in Table 1.

**Table 1:** Transition probability table.

From state	To state	Probability (%)	Source
Healthy	Mild HFMD	0.991	(16, 17)
Healthy	Severe HFMD	0.015	(16, 17)
Severe HFMD	Sequelae	25	(17)
Severe HFMD	Death	3.18	(16)
Severe HFMD	Healthy	71.82	(16, 17)
Healthy	All-cause death	0.371	(14)

### 2.5 Time horizon and model assumptions

The model adopted a cycle length of 1 year. The primary Markov process covered the first 5 years of life, corresponding to the peak incidence period of HFMD and the primary protective window of EV71 vaccination.

Although the acute disease process was modeled during early childhood, outcomes extending beyond childhood were additionally incorporated through lifetime extrapolation methods. Specifically, productivity losses due to premature death were estimated over the projected working lifetime using the human capital approach with annual discounting.

In addition, long-term utility losses associated with EV71-related neurological sequelae were modeled as persistent health outcomes extending beyond the initial 5-year disease horizon. This hybrid time-horizon approach has been applied in previous pediatric vaccine economic evaluations where disease incidence is concentrated in early childhood but some health and economic consequences persist throughout life.

Additional model assumptions were applied in the present analysis. First, herd immunity effects and indirect protection associated with EV71 vaccination were not incorporated into the model. Second, vaccine effectiveness, disease incidence, and transition probabilities were assumed to remain constant throughout the 5-year model horizon. Third, vaccine-induced protection was assumed to persist during the entire follow-up period without waning immunity. Fourth, all newborns entered the model in the healthy state at baseline. Finally, migration and demographic changes within the cohort were not considered during the simulation period.

### 2.6 Study perspective and outcome measures

The analysis was conducted from a societal perspective with limited lifetime extrapolation, incorporating vaccination costs and disease-related direct medical expenditures. Due to data limitations, some broader societal costs, including long-term educational losses and informal caregiving burden associated with neurological sequelae, were not fully quantified.

The main model outputs included: changes in disease burden, total costs, quality-adjusted life years (QALYs), incremental cost-effectiveness ratio (ICER), and benefit–cost ratio (BCR).

By comparing costs and health outcomes across different EV71 vaccination strategies, the study comprehensively evaluated the cost-effectiveness and economic value of a publicly funded vaccination program.

### 2.7 Model parameters

Model parameters included demographic parameters, disease burden parameters, cost parameters, health utility parameters, and vaccine-related parameters. Whenever possible, data were obtained from China or Guangxi-specific sources. In the absence of local data, estimates from regions with similar demographic structures, climatic conditions, and socioeconomic levels were used as proxies. Parameter uncertainty was further assessed through sensitivity analyses.

#### 2.7.1 Demographic parameters

Demographic data were primarily derived from official statistical sources, including the *Guangxi Statistical Yearbook* (12) and the *2024 Statistical Communiqué on National Economic and Social Development of Guangxi* (13). The simulated cohort consisted of all newborns in Guangxi in 2024, with a total population size of 420,000.

The neonatal mortality rate and the mortality rate among children under 5 years of age were obtained from the *2024 Guangxi Health Statistical Communiqué* (14). Population sizes of children under 5 years in Liuzhou and Yulin were estimated based on age-structure data from the Seventh National Population Census combined with city-level statistical yearbooks. These estimates were used to calculate weighted regional medical costs.

#### 2.7.2 Disease burden parameters

Due to the lack of publicly available disease burden data specific to Guangxi, this study used data from neighboring provinces, including Guangdong, Hunan, Guizhou, and Hainan, as proxy estimates. These regions share similar geographic locations, climatic conditions, and socioeconomic characteristics with Guangxi, and exhibit comparable population structures, disease profiles, and healthcare systems.

Although some inter-provincial variability may exist, the use of data from geographically and socioeconomically similar regions is a commonly accepted approach in disease burden studies. This approach ensures the plausibility of parameter estimates and the completeness of the model while maintaining overall analytical validity.

#### 2.7.3 Cost parameters

Direct medical costs included outpatient and inpatient treatment costs for HFMD, as well as long-term treatment costs for sequelae associated with severe HFMD. All direct medical cost data were obtained from real-world hospital information system records collected from maternal and child health hospitals in Liuzhou and Yulin between January 2020 and December 2025. The database included outpatient visits, hospitalization records, and sequelae-related treatment expenditures associated with HFMD. After data cleaning and screening, disease-specific medical costs were extracted and aggregated for economic evaluation. To ensure comparability across different years, all medical costs were adjusted to 2024 price levels using the Chinese Consumer Price Index (CPI) for healthcare expenditure and were subsequently converted to United States dollars (USD) using the average 2024 exchange rate. These data were aggregated using a population-weighted average approach (Equation 1), providing a reliable regional estimate of healthcare resource utilization and economic burden across different disease states. The final database included 6,378 HFMD-related outpatient cases and 524 hospitalization cases from Liuzhou, and 993 outpatient cases and 731 hospitalization cases from Yulin.


$$\begin{array}{l} {\text{C}}_{\text{GuangXi}}=\frac{{\text{C}}_{\text{LiuZhou}}\times {\text{P}}_{\text{LiuZhou}}+{\text{C}}_{\text{YuLin}}\times {\text{P}}_{\text{YuLin}}}{{\text{P}}_{\text{LiuZhou}}+{\text{P}}_{\text{YuLin}}} \end{array}$$ (1)

Specifically, C_Guangxi_ represents the average cost in Guangxi, while C_Liuzhou_ and C_Yulin_ denote the average costs obtained from the respective hospitals. P_Liuzhou_ and P_Yulin_ represent the population of children under 5 years of age in each city.

Vaccine-related parameters were derived from publicly available vaccination inventory data reported by a community health service center in Nanning.

Indirect costs primarily included productivity losses of caregivers. These were estimated as the product of average caregiving duration, the average daily wage, and the number of caregivers per case. The average caregiving duration was calculated based on hospital case databases from Liuzhou and Yulin. The number of caregivers was assumed to be one per hospitalized case. The average daily wage (USD 42.33/day) was derived from the 2024 national average annual wage of employees in urban non-private sectors, as reported by the National Bureau of Statistics of China.

Productivity losses due to premature death in infants were estimated using the human capital approach, based on the national per capita wage income in 2022 (USD 2,987.48/year), a working life expectancy of 42 years, a discount rate of 3%, and an annual wage growth rate of 2%, resulting in a lifetime productivity loss of USD 103,445.07 per death (Equation 2).

Productivity losses due to premature death were estimated using the human capital approach with discounted future earnings:


$$\begin{array}{l} PV=W\times \frac{1-{\left(\frac{1+g}{1+r}\right)}^{n}}{r-g} \end{array}$$ (2)

where W is annual wage income, g is the wage growth rate, r is the discount rate, and n is working life expectancy.

Lifetime productivity losses were discounted at an annual rate of 3% and extrapolated beyond the primary 5-year disease-modeling horizon to reflect long-term societal consequences of premature mortality. All parameter values are summarized in Table 2.

**Table 2:** Model parameters and ranges.

Parameter	Base value	Range	Source
Demographic and epidemiological parameters			
Mortality rate among children under 5 years (per 100,000)	371	-	(14)
Life expectancy (years)	79	-	(15)
HFMD incidence in children under 5 (per 100,000)	4534.8	-	(16)
Proportion of mild cases (%)	99.23	95.0–100	(16)
Proportion of severe cases (%)	0.77	0.6–1.6	(16)
EV71 mild incidence (per 10,000)	990.9	984.6–998.6	(16, 17)
EV71 severe incidence (per 100,000)	15.3	11.94–31.84	(16, 17)
EV71 sequelae incidence (per 100,000)	3.8	2.99–7.96	(16, 17)
Case fatality rate of severe HFMD (%)	3.18	1.48–8.84	(16)
Probability of sequelae after severe HFMD (%)	25%	-	(17)
Proportion of EV-A71 in mild cases (%)	22.02	-	(16)
Proportion of EV-A71 in severe cases (%)	43.88	-	(16)
Proportion of EV-A71 in deaths (%)	74.84	-	(16)
Vaccine parameters			
Coverage of 1-dose vaccination (self-paid) (%)	2.86	2.145–3.055	(16)
Coverage of 2-dose vaccination (self-paid) (%)	10.19	7.6425–12.7375	(16)
Coverage of 1-dose vaccination (NIP) (%)	5	±25	Refer to Group A meningococcal polysaccharide vaccine
Coverage of 2-dose vaccination (NIP) (%)	90	±25	Refer to Group A meningococcal polysaccharide vaccine
Vaccine effectiveness (1 dose) (%)	66.9	45.2–80.0	(18)
Vaccine effectiveness (2 dose) (%)	84.2	80.4–96.3	(18)
Utility parameters			
Utility of mild HFMD	0.78	0.73–0.84	(19)
Utility of severe HFMD	0.61	0.59–0.62	(19)
Utility of sequelae	0.4	0.3–0.5	(20)
Cost parameters (USD)			
Outpatient cost of mild HFMD (per case)	24.03	±25	Real-world data
Hospitalization cost of severe HFMD (per case)	1427.39	±25	Real-world data
Cost of sequelae treatment (per case)	4368.35	±25	Real-world data
Cost per vaccine dose	29.02	±25	Real-world data
Direct non-medical cost (per case)	453.11	±25	(21)
Indirect cost (per case)	345.31	-	(15)
Discount rate (%)	3	0–5	WHO recommends (22)

#### 2.7.4 Health utility parameters

Health utility values were assigned to each health state to estimate QALYs. Utility values for mild HFMD and severe HFMD were obtained from Zheng et al. using EQ-5D measurements among Chinese pediatric HFMD patients (17, 18). Because China-specific utility estimates for EV71-related neurological sequelae remain limited, the utility value for sequelae was derived from Koh et al (20), which evaluated long-term neurological impairment associated with HFMD in Asian pediatric populations. The sequelae state was assumed to represent persistent long-term neurological impairment.

### 2.8 Economic evaluation

All analyses were conducted using R software (version 4.4.3). Both cost-effectiveness analysis (CEA) and cost–benefit analysis (CBA) were performed to evaluate the economic value of including the EV71 vaccine in the National Immunization Program (NIP).

#### 2.8.1 Cost-effectiveness analysis

Cost-effectiveness analysis used quality-adjusted life years (QALYs) as the primary health outcome (Equation 3). The incremental cost-effectiveness ratio (ICER) was calculated as:


$$\begin{array}{l} \text{ICER}=\frac{C_{\text{NIP}}-C_{\text{noNIP}}}{E_{\text{NIP}}-E_{\text{noNIP}}} \end{array}$$ (3)

where C_NIP_ and C_noNIP_ represent total costs under the NIP and non-NIP scenarios, respectively, and E_NIP_ and E_noNIP_ denote total QALYs. Both costs and health outcomes were discounted at an annual rate of 3%, consistent with recommendations from international pharmacoeconomic evaluation guidelines and previous vaccine economic evaluations (23). Although the primary disease-modeling horizon was limited to 5 years, discounting remained necessary because the analysis incorporated long-term outcomes extending beyond childhood, including productivity losses associated with premature mortality and long-term utility losses related to neurological sequelae.

Following World Health Organization (WHO) recommendations, the willingness-to-pay (WTP) threshold was defined based on per capita gross domestic product (GDP): interventions with an ICER below one times GDP per capita were considered highly cost-effective; those between one and three times GDP per capita were considered cost-effective; and those above three times GDP per capita were considered not cost-effective.

#### 2.8.2 Cost–benefit analysis

Cost–benefit analysis was conducted using the benefit–cost ratio (BCR) (Equation 4), defined as:


$$\begin{array}{l} BCR=\frac{\text{P}{\text{V}}_{\left(\text{Total Benefit Relative to Control}\right)}}{\text{P}{\text{V}}_{\left(\text{Total cost relative to Control}\right)}}=\frac{\sum_{t=0}^{T}\frac{B_{t}}{{\left(1+r\right)}^{t}}}{\sum_{t=0}^{T}\frac{C_{t}}{{\left(1+r\right)}^{t}}} \end{array}$$ (4)

where B_t_ represents monetized health benefits, C_t_ represents incremental costs in year t, r is the discount rate, and T is the time horizon (5 years).

A BCR greater than 1 indicates that the economic benefits of the intervention exceed its costs, suggesting economic feasibility.

#### 2.8.3 Sensitivity analysis

One-way sensitivity analysis was performed to assess the robustness of the model results. Key parameters were varied individually across predefined ranges while holding other parameters constant. The impact of parameter variation on ICER and BCR was evaluated.

Parameters included in the sensitivity analysis comprised disease incidence, vaccine effectiveness, cost parameters, and vaccination coverage rates.

#### 2.8.4 Probabilistic sensitivity analysis

Probabilistic sensitivity analysis (PSA) was conducted to evaluate the joint uncertainty of model parameters. Monte Carlo simulation with 10,000 iterations was performed by simultaneously varying key parameters according to predefined probability distributions. Beta distributions were assigned to probabilities, utilities, and vaccine coverage parameters; gamma distributions were assigned to cost parameters; and log-normal distributions were assigned to relative risks and vaccine effectiveness parameters.

The PSA results were used to generate cost-effectiveness scatter plots and cost-effectiveness acceptability curves (CEACs), illustrating the probability that the EV71 vaccination strategy would be considered cost-effective under different willingness-to-pay thresholds.

## 3 Results

### 3.1 Base-case analysis

A decision tree–Markov model was used to compare the health and economic outcomes of including the EV71 vaccine in the NIP vs. maintaining the current self-paid strategy. The model simulated a birth cohort of 420,000 newborns over a 5-year time horizon with a discount rate of 3%.

Under the NIP scenario, the government fully funded vaccination, providing two doses of EV71 vaccine free of charge. Under the self-paid strategy, vaccination decisions were made by households.

The model estimated that cumulative vaccination coverage increased from 54,810 individuals under the self-paid strategy to 399,000 individuals under the NIP scenario, representing an increase of 344,190 vaccinated individuals.

### 3.2 Cost–benefit analysis (CBA)

Based on a reported vaccine price of USD 29.02 per dose, the total vaccination cost under the self-paid strategy was estimated at USD 2.83 million, compared with USD 22.55 million under the NIP scenario, resulting in an incremental vaccination cost of USD 19.71 million.

As shown in Table 3, inclusion of EV71 vaccination in the NIP was projected to prevent 14,234 HFMD cases, including approximately 13,949 mild cases and 215 severe cases. Compared with the non-NIP scenario, direct medical costs, direct non-medical costs, indirect costs, and productivity losses decreased by approximately 76%.

**Table 3:** Cost–benefit analysis results of EV71 vaccination strategies.

Cost Category	NIP (USD million)	Non-NIP (USD million)	NIP vs. NonNIP	
			Increment (USD million)	Change (%)
Vaccination cost	2254.66	283.23	1971.42	696.04
Disease-related cost	313.64	1328.43	−1014.79	−76.39
Mild case treatment cost	10.36	43.87	−33.52	−76.39
Severe case treatment cost	9.50	40.25	−30.74	−76.39
Sequelae treatment cost	7.22	30.59	−23.37	−76.39
Direct medical cost	27.08	114.71	−87.63	−76.39
Direct non-medical cost	3.02	12.78	−9.76	−76.39
Indirect cost	2.30	9.74	−7.44	−76.39
Productivity loss	281.24	1191.21	−909.97	−76.39

Overall, total HFMD-related costs decreased by USD 10.15 million, while vaccination costs increased by USD 19.71 million, resulting in a BCR of 0.515.

### 3.3 Cost-effectiveness analysis (CEA)

Under the self-paid scenario, the model estimated 21 HFMD-related deaths. Inclusion of the vaccine in the NIP reduced the number of deaths to 5, preventing 16 deaths (a 76.39% reduction).

Similarly, total HFMD cases decreased by 14,234 (a 76.39% reduction). The ICER per death averted was USD 635,800, and the ICER per case averted was USD 712.92.

In terms of health outcomes, the NIP strategy resulted in an additional gain of 1,260.8 QALYs, with an ICER of USD 8,319.99 per QALY. Compared with the per capita GDP of Guangxi in 2024 (USD 8,280.31), the ICER was approximately 1.005 times GDP per capita, suggesting that the intervention yields acceptable cost-effectiveness per WHO thresholds.

### 3.4 Sensitivity analysis

**Figure 2:**
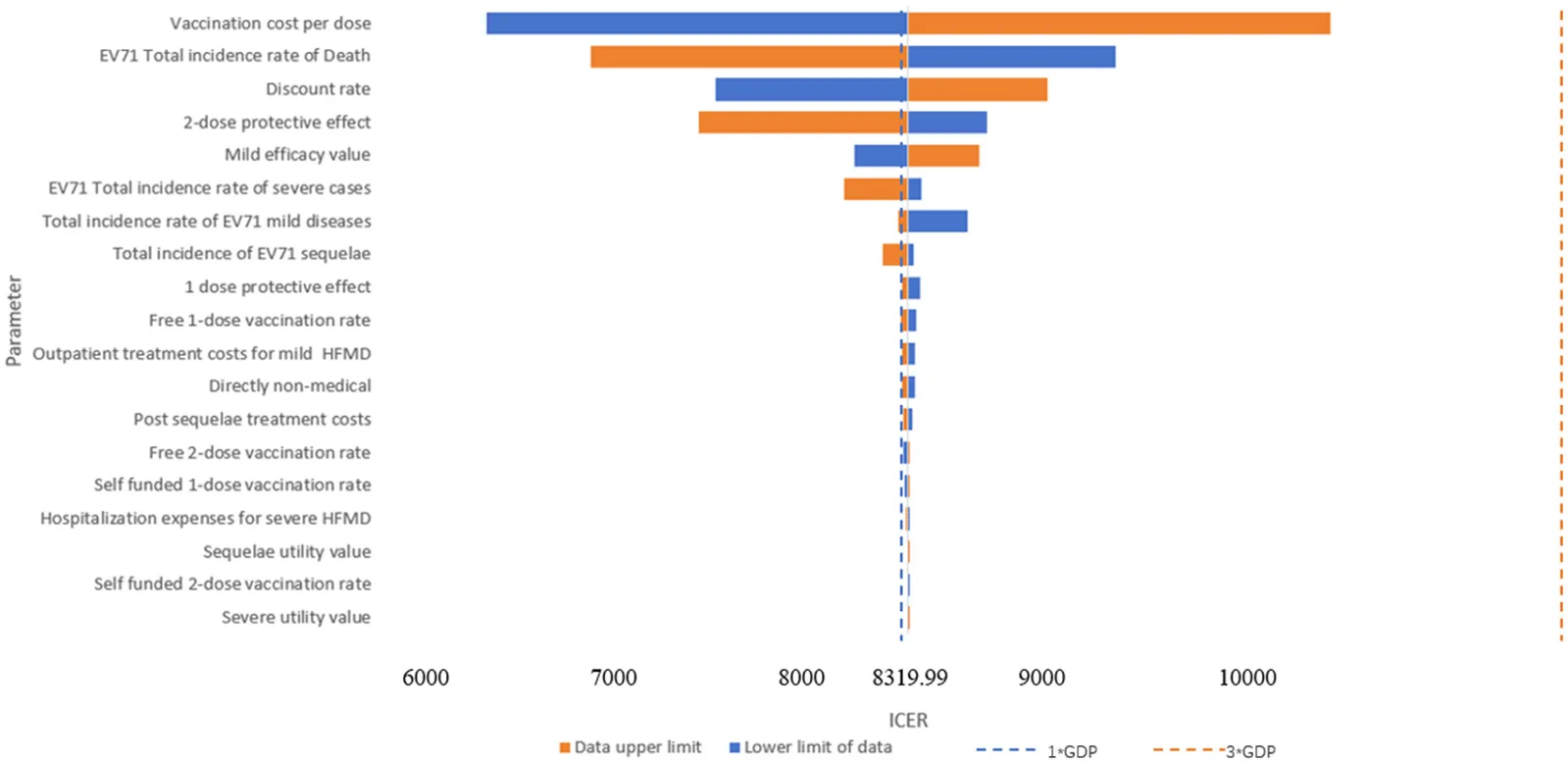
Results of the one-way sensitivity analysis.

The results of the one-way sensitivity analysis are presented in Figure 2. Several parameters influenced the ICER, with the vaccine price per dose identified as the most influential factor (Table 4).

**Table 4:** Sensitivity analysis results.

Parameter	ICER of Lower limit of data	ICER of Data upper limit
Severe utility value	8318.316	8320.828
Self funded 2-dose vaccination rate	8321.902	8317.956
Sequelae utility value	8315.911	8324.075
Hospitalization expenses for severe HFMD	8328.721	8311.259
Self funded 1-dose vaccination rate	8299.501	8325.7
Free 2-dose vaccination rate	8295.944	8326.882
Post sequelae treatment costs	8342.04	8297.941
Directly non-medical	8349.477	8290.504
Outpatient treatment costs for mild HFMD	8351.615	8288.366
Free 1-dose vaccination rate	8356.504	8284.355
1 dose protective effect	8375.398	8286.826
Total incidence of EV71 sequelae	8343.978	8197.357
Total incidence rate of EV71 mild diseases	8607.628	8269.998
EV71 Total incidence rate of severe cases	8384.62	8008.4
Mild efficacy value	8058.823	8660.989
2-dose protective effect	8697.414	7312.686
Discount rate	7389.558	8990.408
EV71 Total incidence rate of Death	9317.46	6793.893
Vaccination cost per dose	6290.52	10349.461

When the price of each vaccine dose drops to USD28.95, ICER is equivalent to once the GDP, making it highly cost-effective; When the price of each dose of the vaccine rises to USD 61.54, ICER is equivalent to three times GDP and therefore does not have cost-effectiveness. When the vaccine price drops to USD15.44, the BCR is 1, and the cost paid is equal to the profit obtained. Further price reduction can result in net profit.

Other key parameters included the case fatality rate of severe EV71 infection, the discount rate, the effectiveness of the two-dose vaccination schedule, the utility value of mild HFMD, and the incidence of severe EV71 infection.

Parameter variation analysis showed that increases in vaccine price, discount rate, utility values for mild disease, vaccination coverage under the self-paid one-dose scenario, and utility values for severe disease were associated with higher ICER values, thereby reducing cost-effectiveness.

### 3.5 Probabilistic sensitivity analysis

Probabilistic sensitivity analysis demonstrated that the inclusion of EV71 vaccination in the National Immunization Program remained economically favorable under most simulation scenarios.

**Figure 3:**
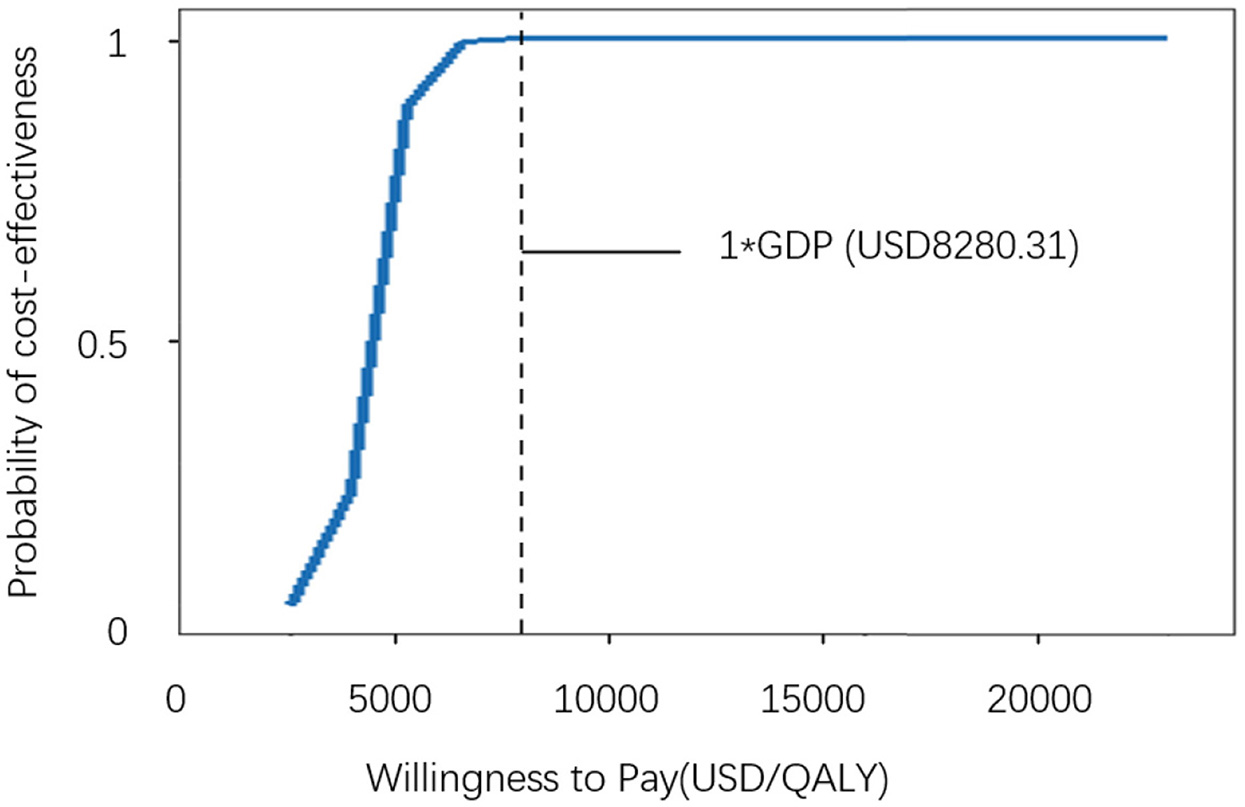
Cost-effectiveness acceptability curve (CEAC).

**Figure 4:**
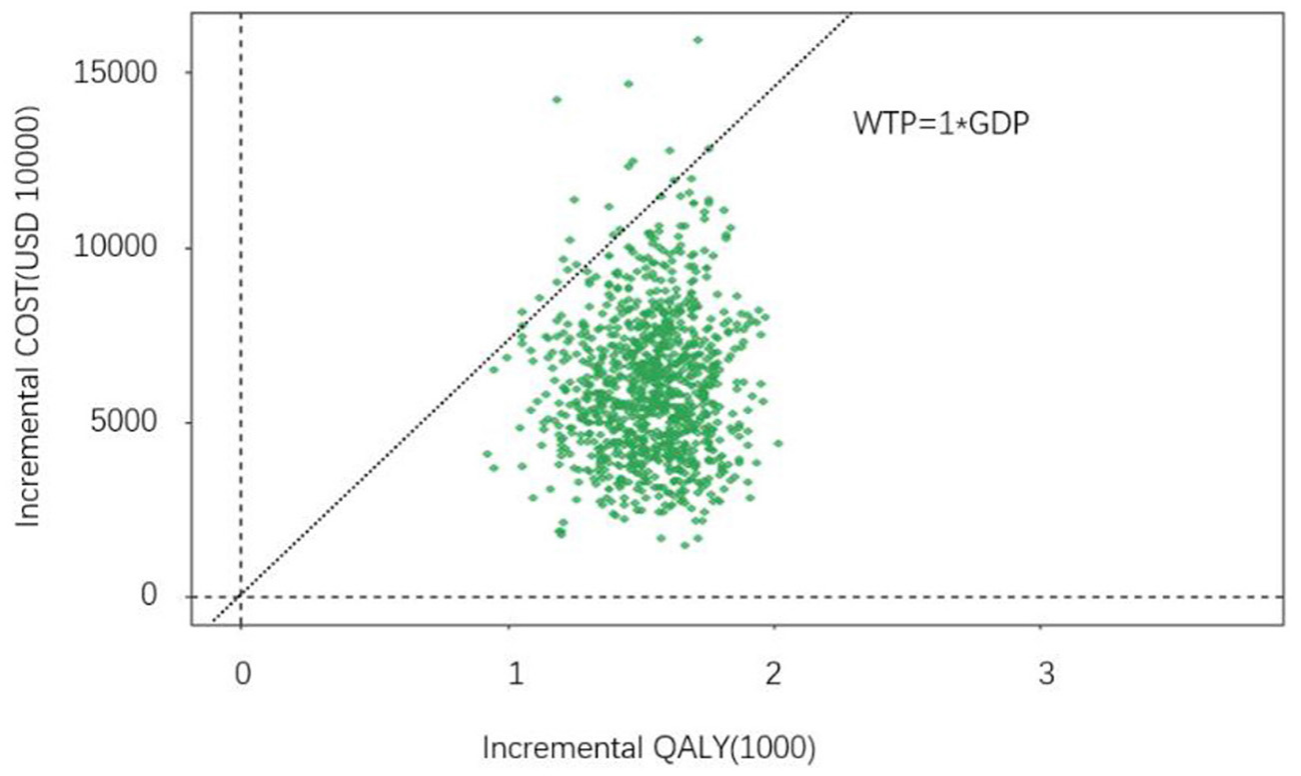
Probabilistic sensitivity analysis (PSA).

The cost-effectiveness acceptability curve indicated that the probability of cost-effectiveness increased progressively with higher willingness-to-pay thresholds (Figure 3). The PSA scatter plot showed that most incremental cost-effectiveness pairs were distributed in the northeast quadrant, indicating increased health benefits accompanied by higher costs (Figure 4).

## 4 Discussion

This study employed a decision tree–Markov hybrid model to evaluate the health and economic impact of including the EV71 vaccine in the National Immunization Program (NIP) in Guangxi, China, from a societal perspective. The findings indicate that, compared with the current self-paid vaccination strategy, inclusion in the NIP would substantially reduce the burden of hand, foot, and mouth disease (HFMD) and achieve acceptable cost-effectiveness under the predefined willingness-to-pay (WTP) threshold. These findings are generally consistent with previous economic evaluations of EV71 vaccination in China (24, 25). However, important quantitative differences were observed. In the present study, the estimated ICER was USD 8,319.99 per QALY, whereas Wang et al. (24) reported substantially higher ICERs ranging from USD 49,372 to USD 69,260 per QALY, exceeding the three-times GDP threshold and indicating that the vaccination strategy was not cost-effective under their baseline assumptions. Wang et al. further demonstrated that reducing vaccine prices below USD 14.6 per dose or USD 8.3 per dose would make the program cost-effective or highly cost-effective, respectively. Notably, our threshold analysis identified a similar price range, with a break-even price of approximately USD 14.94 per dose at which the benefit–cost ratio (BCR) reached 1. The more favorable economic results observed in our study may be explained by differences in vaccine financing assumptions, regional disease burden, healthcare costs, vaccination coverage under NIP implementation, and the use of Guangxi-specific real-world cost data. These findings reinforce vaccine pricing and financing mechanisms as central determinants of the economic value of EV71 vaccination.

A key finding of this study is that vaccination financing strongly influences health outcomes. Under the self-paid model, vaccination coverage is constrained by household affordability, whereas inclusion in the NIP substantially increased coverage in the model. This is consistent with empirical evidence from China showing that public financing significantly improves vaccine uptake, particularly in economically disadvantaged regions (26, 27). As a result, increased coverage was associated with a reduction of more than 70% in both HFMD cases and deaths, consistent with clinical and modeling evidence of high vaccine effectiveness against severe EV71 infection (28–30). Because herd immunity effects were not incorporated, these estimates are likely conservative, as EV71 vaccination may reduce transmission and provide indirect protection to unvaccinated individuals (31).

From an economic perspective, although NIP inclusion increases vaccination program costs, these are partially offset by substantial reductions in direct medical costs, non-medical costs, and productivity losses. Overall disease-related costs decreased by approximately 76%. However, the estimated benefit–cost ratio (BCR) was 0.515, indicating that under current vaccine pricing conditions, monetized benefits do not fully offset program costs. This suggests that, at present, EV71 vaccination under NIP conditions is not cost-saving from a strict cost–benefit perspective.

Nevertheless, interpretation of this result should consider complementary economic frameworks. While cost–benefit analysis evaluates net monetary returns, cost-effectiveness analysis assesses health gains relative to willingness-to-pay thresholds. In this study, the ICER was USD 8,319.99 per QALY, approximately 1.005 times the per capita GDP of Guangxi in 2024, indicating that the intervention remains cost-effective under commonly used GDP-based criteria (22). Therefore, despite a BCR below 1, EV71 vaccination still represents an economically justifiable intervention in terms of health gain efficiency.

Sensitivity analyses further confirmed the robustness of the findings. A probabilistic sensitivity analysis using Monte Carlo simulation (10,000 iterations) demonstrated consistent results, and the cost-effectiveness acceptability curves (CEACs) indicated a high probability of cost-effectiveness across a wide range of willingness-to-pay thresholds. Among all parameters, vaccine price remained the most influential determinant of both ICER and BCR outcomes, followed by disease incidence, vaccine effectiveness, and severe case fatality rate.

Threshold analysis highlighted vaccine price as the primary policy lever. The break-even price at which BCR equals 1 was estimated at approximately USD 14.94 per dose. Importantly, this value appears achievable under China's existing vaccine procurement system. Provincial vaccine alliance purchasing mechanisms for vaccines have achieved documented price reductions of approximately 40%−55%, and up to 60% for selected vaccines under volume-based procurement arrangements (32). These established policy instruments provide a realistic institutional pathway for reducing EV71 vaccine prices toward the identified threshold. In addition to price effects, increases in disease incidence, vaccine effectiveness, or vaccination coverage would further enhance both cost-effectiveness and cost–benefit performance.

Broader evidence supports the economic value of vaccination strategies in similar settings. Systematic reviews have shown that vaccination programs in low- and middle-income countries often generate substantial health and economic returns (33). Consistently, studies in China have demonstrated favorable cost-effectiveness for vaccines such as the 13-valent pneumococcal conjugate vaccine (PCV13) in high-burden regional immunization programs (34). These findings strengthen the external validity of our results.

Several limitations should be acknowledged. First, some model parameters were derived from literature or proxy data from neighboring provinces due to limited Guangxi-specific epidemiological data availability. Second, the model did not incorporate dynamic transmission effects such as herd immunity or serotype variation, which may lead to underestimation of long-term benefits. Third, although lifetime extrapolation was applied for mortality and sequelae-related outcomes, the model does not fully capture long-term disease progression trajectories. In addition, broader societal consequences of HFMD sequelae, including educational loss, caregiver burden, psychosocial impact, and social care costs, were not fully quantified, suggesting that the overall economic benefits of vaccination may be underestimated.

## 5 Conclusions

In conclusion, under current model assumptions, inclusion of the EV71 vaccine in the National Immunization Program would substantially reduce the burden of HFMD among children and is considered cost-effective. Vaccine price reduction plays a critical role in improving economic outcomes, and appropriate pricing strategies could enable the intervention to achieve net economic benefits. This study provides robust quantitative evidence to support regional vaccine policy decision-making.

## Data Availability

The original contributions presented in the study are included in the article/supplementary material, further inquiries can be directed to the corresponding authors.
