# Correction: Economic evaluation of including the EV71 vaccine in the national immunization program: a modeling study in Guangxi, China

**DOI:** 10.3389/fpubh.2026.1951313

**Published:** 2026-08-26

**Authors:** Quan He, Xiaoting Liao, Linlin Deng, Zhengqin Su, Weiwen Zhou, Yi Mo, Xiong Zou, Caifang Li, Yong Lv, Caitong He, Haibin Wei, Hai Li

**Affiliations:** 1 School of Public Health and Management, Guangxi University of Traditional Chinese Medicine, Nanning, China; 2 Key Laboratory of Integrated Traditional Chinese and Western Medicine Translational Medicine for High Incidence Infectious Diseases in Guangxi, Nanning, China; 3 Sterile Supply Department, The First Affiliated Hospital of Guangxi University of Traditional Chinese Medicine, Nanning, China; 4 Immunization Planning Institute, Guangxi Zhuang Autonomous Region Center for Disease Control and Prevention, Nanning, China; 5 Vaccine Clinical Research Institute, Guangxi Zhuang Autonomous Region Center for Disease Control and Prevention, Nanning, China; 6 The Maternal and Child Health Hospital of Guangxi Zhuang Autonomous Region, Nanning, China; 7 Department of Pediatrics, Liuzhou Maternal and Child Health Hospital, Liuzhou, China; 8 Yulin Maternal and Child Health Hospital, Yulin, China; 9 Development Planning Department, Guangxi University of Traditional Chinese Medicine, Nanning, China

**Keywords:** cost–benefit analysis, cost-effectiveness analysis, decision tree–Markov model, EV71, health economics, HFMD, national immunization program, vaccination policy

There was a mistake in Figure 3 as published. The wrong image file was inadvertently used during the preparation of the final manuscript files. The corrected Figure 3 appears below.

**Figure 3:**
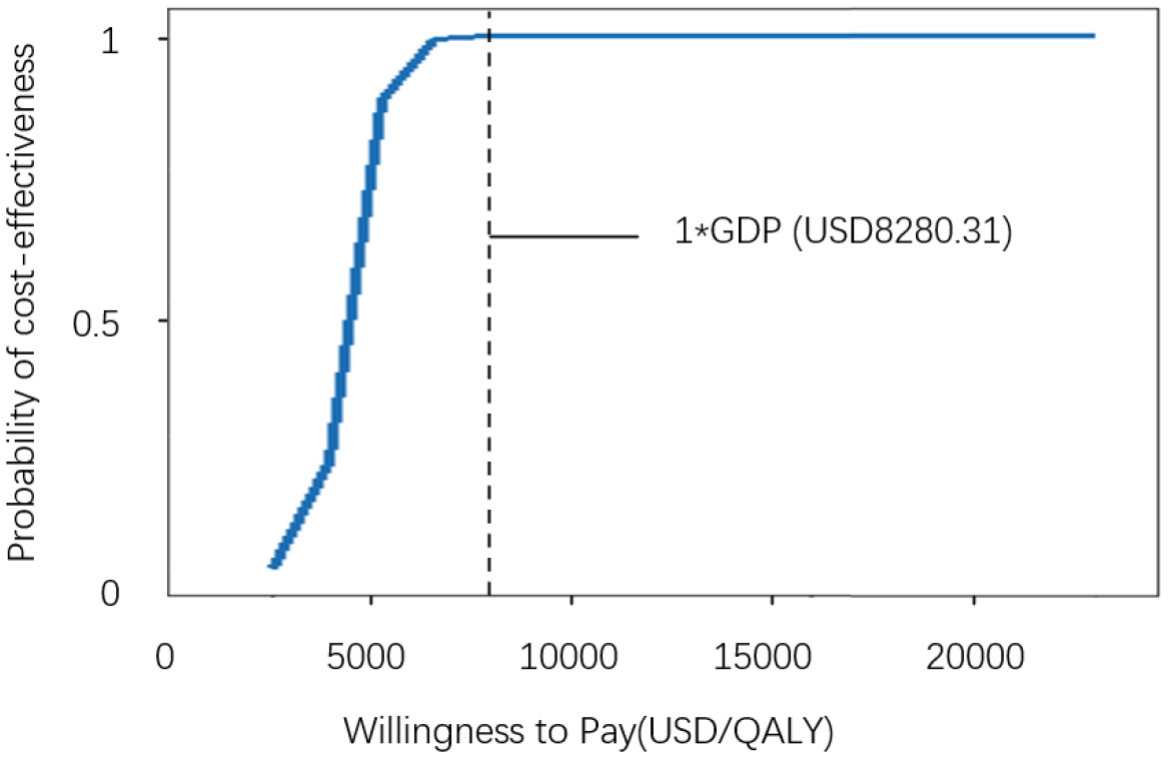
Cost-effectiveness acceptability curve (CEAC).

There was a mistake in Figure 4 as published. The wrong image file was inadvertently used during the preparation of the final manuscript files. The corrected Figure 4 appears below.

**Figure 4:**
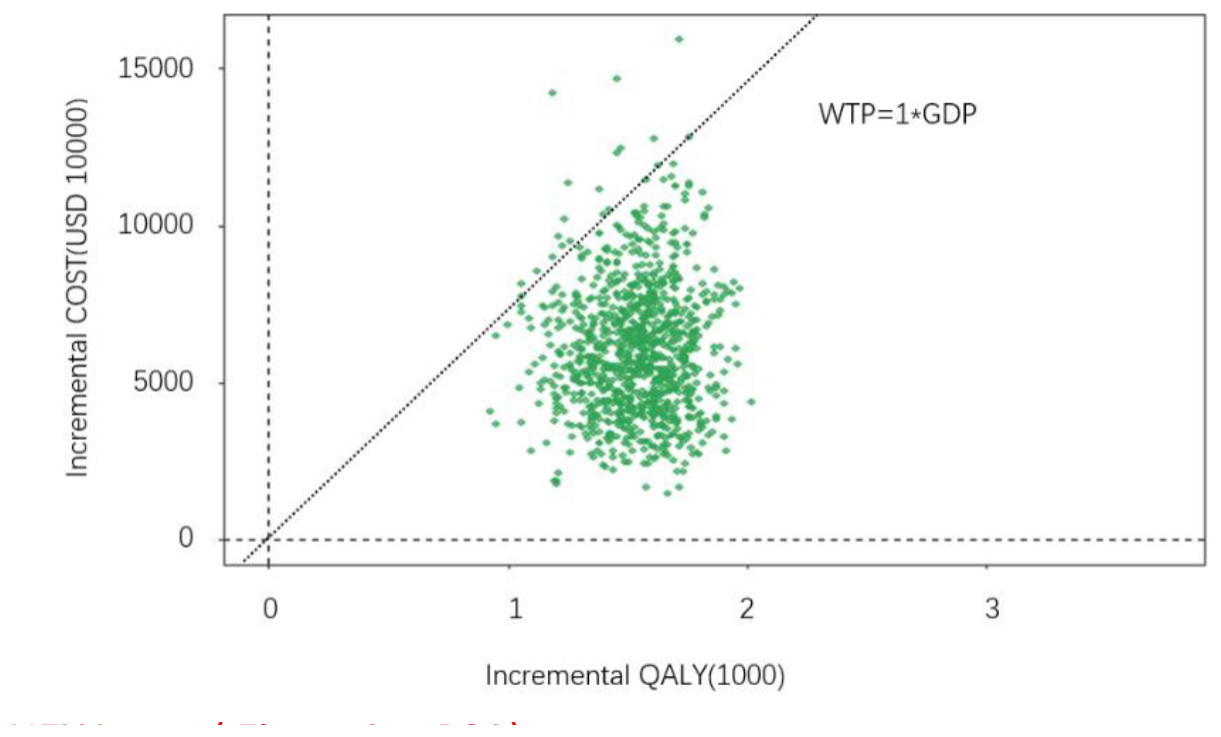
Probabilistic sensitivity analysis (PSA).

The original version of this article has been updated.

